# Preceding Host History of Conjugative Resistance Plasmids Affects Intra- and Interspecific Transfer Potential from Biofilm

**DOI:** 10.1128/msphere.00107-23

**Published:** 2023-04-05

**Authors:** Ilmur Jonsdottir, Cindy Given, Reetta Penttinen, Matti Jalasvuori

**Affiliations:** a Department of Biological and Environmental Science, Nanoscience Center, University of Jyväskylä, Jyväskylä, Finland; b Department of Biology, University of Turku, Turku, Finland; University of Michigan-Ann Arbor

**Keywords:** antibiotic resistance, biofilms, evolutionary rescue, experimental evolution, horizontal gene transfer (HGT), host-plasmid interactions, plasmids

## Abstract

Conjugative plasmids can confer antimicrobial resistance (AMR) to their host bacterium. The plasmids disperse even between distantly related host species, rescuing the host from otherwise detrimental effects of antibiotics. Little is known about the role of these plasmids in the spread of AMR during antibiotic treatment. One unstudied question is whether the past evolutionary history of a plasmid in a particular species creates host specificity in its rescue potential or if interspecific coevolution can improve interspecific rescues. To study this, we coevolved the plasmid RP4 under three different host settings; solely Escherichia coli or Klebsiella pneumoniae, or alternating between both of them. The ability of evolved plasmids in bacterial biofilm to rescue susceptible planktonic host bacteria of either the same or different species during beta-lactam treatment was tested. The interspecific coevolution seemed to decrease rescue potential for the RP4 plasmid, while the K. pneumoniae evolved plasmid became more host specific. Large deletion in the region encoding the mating pair formation (Tra2) apparatus was detected in the plasmids evolved with K. pneumoniae. This adaptation resulted in the exapted evolution of resistance against a plasmid-dependent bacteriophage PRD1. Further, previous studies have suggested that mutations in this region completely abolish the plasmid’s ability to conjugate; however, our study shows it is not essential for conjugation but rather affects the host-specific conjugation efficiency. Overall, the results suggest that previous evolutionary history can result in the separation of host-specific plasmid lineages that may be further amplified by unselected exaptations such as phage resistance.

**IMPORTANCE** Antimicrobial resistance (AMR) is a major global public health threat which can rapidly spread in microbial communities via conjugative plasmids. Here, we advance with evolutionary rescue via conjugation in a more natural setting, namely, biofilm, and incorporate a broad-host range plasmid RP4 to test whether intra- and interspecific host histories affect its transfer potential. Escherichia coli and Klebsiella pneumoniae hosts were seen to elicit different evolutionary influences on the RP4 plasmid, leading to clear differences in the rescue potential and underlining the significant role of the plasmid-host interactions in the spread of AMR. We also contradicted previous reports that established certain conjugal transfer genes of RP4 as essential. This work enhances the understanding of how plasmid host range evolve in different host settings and further, the potential effects it may have on the horizontal spread of AMR in complex environments such as biofilms.

## INTRODUCTION

Plasmids are self-replicating extrachromosomal genetic elements of bacteria. Conjugative plasmids are able to encode a bridge between their current host and suitable neighboring bacteria, allowing for horizontal gene transfer (HGT) via conjugation (1, 2). These plasmids are part of the antimicrobial resistance (AMR) global health problem as they can harbor and confer AMR genes (3–11). Plasmids and bacteria are intrinsically linked through their shared environment (12–15). Through conjugation certain plasmids can spread intra- and interspecifically depending on their host-range (16, 17). However, plasmids carry a fitness-cost that is often associated with their maintenance in the host cell. Compensatory mutations within the host chromosome and the plasmid can alleviate the plasmid fitness cost to help promote their persistence in the community (18–27). Nevertheless, the long-term survival of plasmids in communities remains puzzling due to their costs to the host (sometimes referred as “the plasmid-paradox”) (28).

Biofilms are one microbial formation where plasmids are maintained (29, 30). The individuals of these connected ecosystems interact more frequently with their neighbors, allowing for better mating pair formation and ultimately higher chance of plasmid transfer (31–34). Recently, biofilms were shown to improve the persistence of AMR plasmids (25, 35). Worryingly, plasmid-carrying resistant bacteria can save susceptible cells in their vicinity via HGT even after exposure to antibiotics (36, 37). However, this so-called evolutionary rescue via HGT has not been studied for biofilm associated bacteria despite the ubiquity of biofilms in nature.

The overall survival and success of all plasmids are influenced by “built-in” evolutionary trade-offs in a multihost environment (38, 39). Namely, natural selection within a single host strain allows plasmids to coevolve with their hosts and compensate any detrimental plasmid fitness effects (23, 26, 40). However, a long-term adaptation to a specific host can increase the fitness cost of the plasmid in less similar hosts as these changes are only continuously checked against that particular within-host environment (41). The adaptive changes in one host may cause conflicts (on a molecular level) in others, similar to speciation in sexually reproducing organisms (38, 42).

Alternatively, plasmids that regularly change host species are likely to maintain lower fitness effects in all their regularly “evaluated” hosts, as well as more likely to be devoid of specific adaptations that help in one host but cause conflicts in others (38, 43). Therefore, initially homogenous plasmid population could diverge to “host-generalists” and “host-specialists.” To what extent this occurs, is still unclear. Without strong selection for any particular host species, the existence of such plasmid groups in a community may be negligible. However, in specific situations the preceding host history may become relevant. For example, sudden change in environmental conditions (such as administration of antibiotics) can favor different subpopulations of plasmids that may have adapted to their current host species, to multiple species, or to a specific alternative species. Further, the plasmid donor species may play a vital role as conjugation intra- and interspecifically may affect the transfer rate of the plasmid to sensitive hosts.

**FIG 1:**
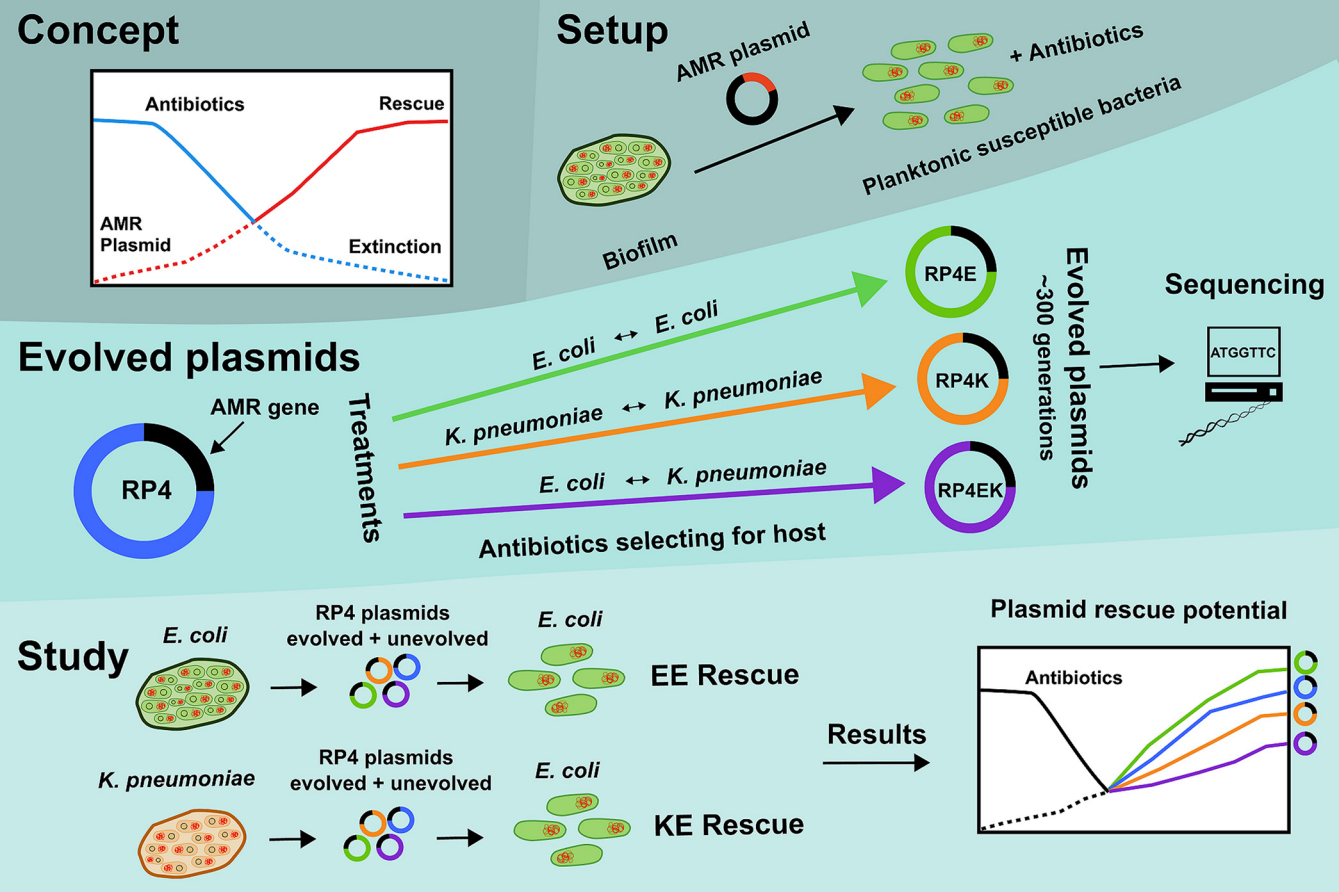
Schematic design of this study. The key concepts in this experimental design were host history and evolutionary rescue via HGT. The rescue occurred from biofilm, rescuing planktonic antibiotic-susceptible bacteria after 1-h antibiotic exposure. The plasmids used in the rescue were RP4 and variants of RP4 after 300 generations of coevolution with E. coli (RP4E), K. pneumoniae (RP4K) or alternating between them both (RP4EK). Two rescue setups were performed; intraspecific: E. coli rescuing E. coli (EE) and interspecific: K. pneumoniae rescuing E. coli (KE), with the results measuring the rescue potential of each plasmid.

We aimed to better understand the potential of plasmids on rescuing susceptible bacteria from the effects of lethal antibiotics. To determine the factors that affect the rescue potential, we utilized plasmids with different characteristics and different evolutionary histories (Fig. 1). We hypothesized that a plasmid that was coevolved intraspecifically with its host (E. coli or K. pneumoniae) would exhibit host specificity in its rescue and a plasmid with a history of interspecific coevolution (between E. coli and K. pneumoniae) would broaden its rescue prospects. We observed a clear difference in the plasmid adaptation with a stronger evolutionary response linked to K. pneumoniae, which resulted in a major deletion of RP4’s mating pair formation gene core (Tra2). Without the transfer genes the rescue potential of the plasmid decreased but did not dissipate entirely and conferred plasmid-dependent phage (PRD1) resistance.

## RESULTS

### Rescue potential differs between plasmids of different characteristics.

**FIG 2:**
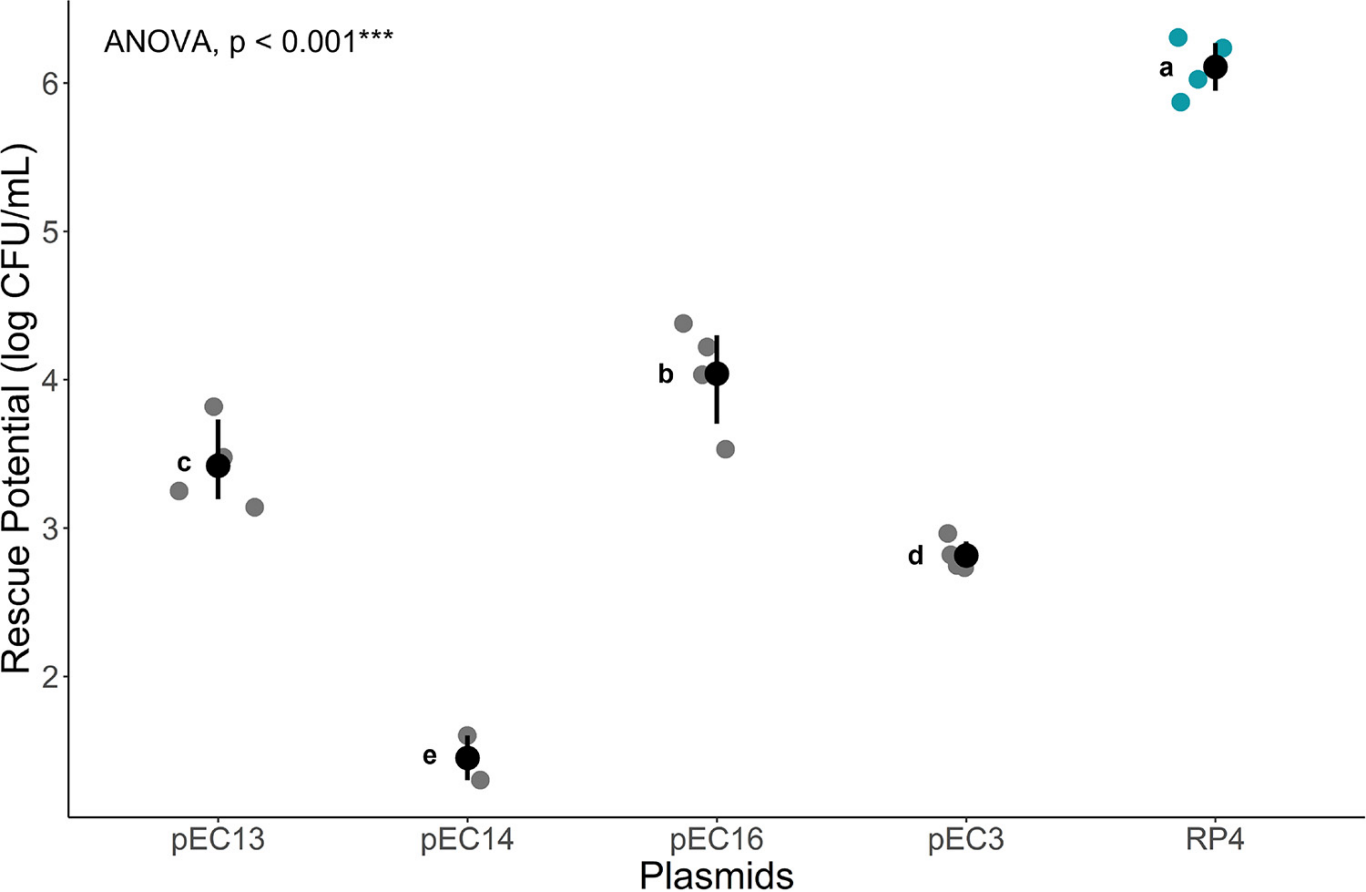
Evolutionary rescue potential of pEC(3,13,14,and 16) (gray) and RP4 plasmid (blue) in E. coli from biofilm to susceptible planktonic bacteria. Rescue potential was measured as the conjugation rate (CFU/mL) of each plasmid (N = 4). Two replicates for pEC14 did not produce any transconjugants and were omitted from this figure. The mean and bootstrap confidence interval of each plasmid are represented by point ranges. A one-way ANOVA with Tukey-HSD post hoc was performed between all plasmid-carrying strains. The P-value is shown, and the Tukey’s HSD letters (a-e) next to each point range indicate whether there is a statistical difference.

We sought out to investigate if different genotypic characteristics of plasmids effected their rescue potential by testing the rescue potential of six plasmids harboring separate features in the same rescue setup (E. coli to E. coli; EE). The density of rescued cells varied across 4 orders of magnitude (Fig. 2). Plasmid pEC15 was unable to rescue any sensitive hosts and was therefore omitted from the subsequent analysis. Each plasmid in the evolutionary rescue experiment showed statistically significant differences in their rescue potential (ANOVA; Tukey-HSD, *P* < 0.001). The plasmid pEC14 carried the lowest rescue potential aside from pEC15, with two of the replicates for pEC14 omitted as they did not rescue any planktonic cells. RP4 plasmid showed the highest rescue potential.

### Past evolutionary history with K. pneumoniae significantly affects the rescue potential of RP4 plasmid.

**FIG 3:**
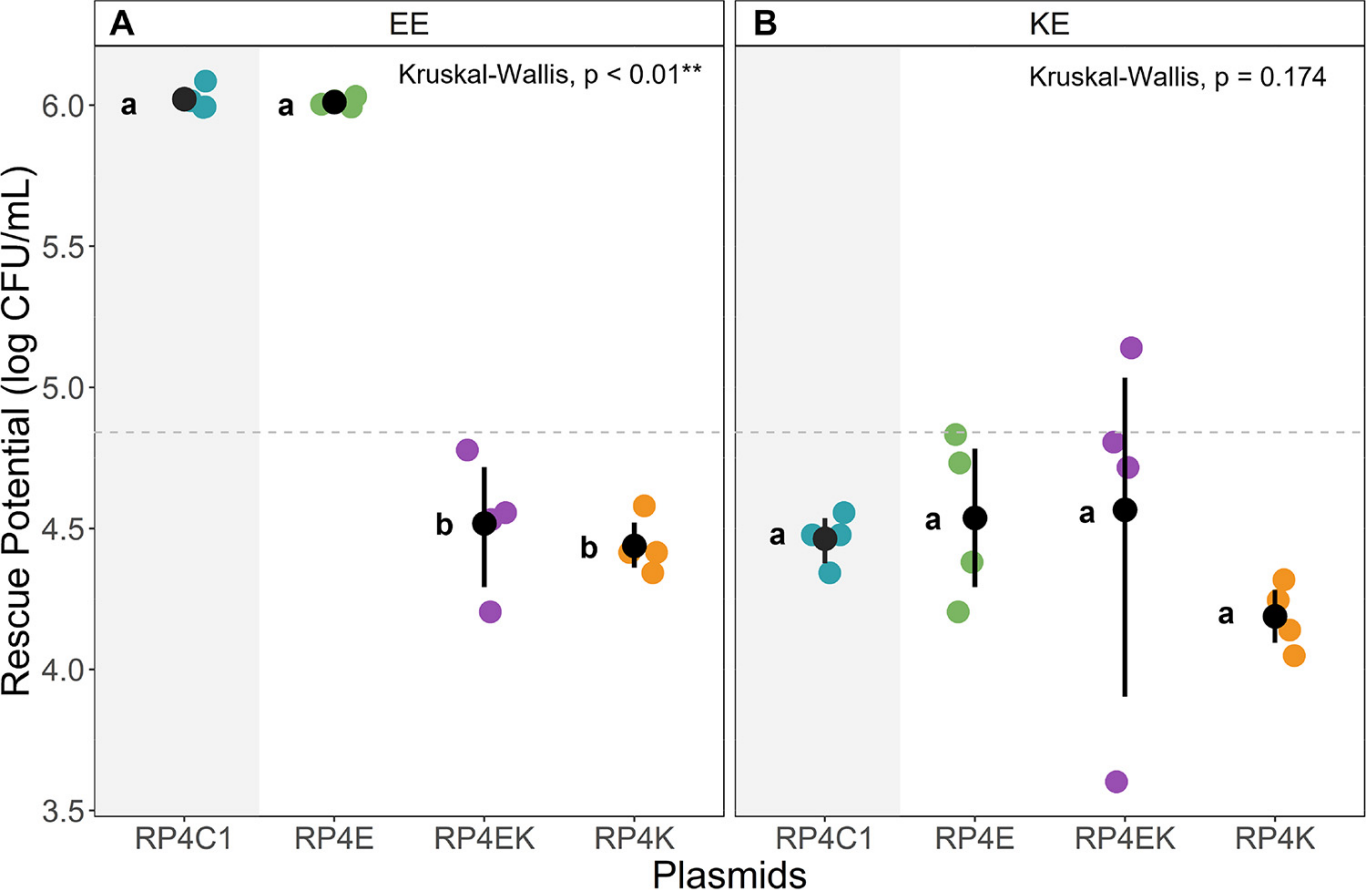
Evolutionary rescue potential of evolved RP4 plasmids (RP4E; green, RP4EK; purple, RP4K; orange) and the unevolved RP4 (RP4C1; blue) from biofilm to susceptible planktonic bacteria in: (A) E. coli to E. coli (EE), and (B) K. pneumoniae to E. coli (KE) rescue setups. Rescue potential was measured as the conjugation rate (CFU/mL) of each plasmid (N = 4) in the rescue setup. The mean and bootstrap confidence interval of each plasmid can be seen represented by point ranges. A Kruskal-Wallis with Dunn test for post hoc was performed between all plasmid-carrying strains. The P-value is shown, and the letters (a-e) next to each point range indicate whether there was a statistical difference between the plasmids found in the Dunn test. The mean of the entire data can be seen represented in the dashed line.

In the E. coli to E. coli (EE) evolutionary rescue setup, the E. coli plasmid (RP4E) had the same rescue potential as the unevolved plasmid (RP4C1) (Fig. 3A). However, the rescue potential significantly decreased with the K. pneumoniae evolved plasmid (RP4K) and the interspecific evolved plasmid (RP4EK), with both evolved plasmids having similar mean rescue potentials. This suggests that evolutionary history involving K. pneumoniae decreased RP4 rescue potential between biofilm-associated and planktonic E. coli (Kruskal-Wallis; Dunn, *P* < 0.01). Further, K. pneumoniae as a plasmid donor had significantly less potential in rescuing planktonic E. coli regardless of the past host-history. This is seen clearly in the K. pneumoniae to E. coli (KE) evolutionary rescue setup, in which all plasmids (RP4C1, RP4E, RP4K, RP4EK) give similar mean rescue potential with no statistical difference (Kruskal-Wallis; Dunn, *P* = 0.174) (Fig. 3B).

### Intraspecific coevolution with K. pneumoniae caused significant host specificity of RP4 plasmid.

**FIG 4:**
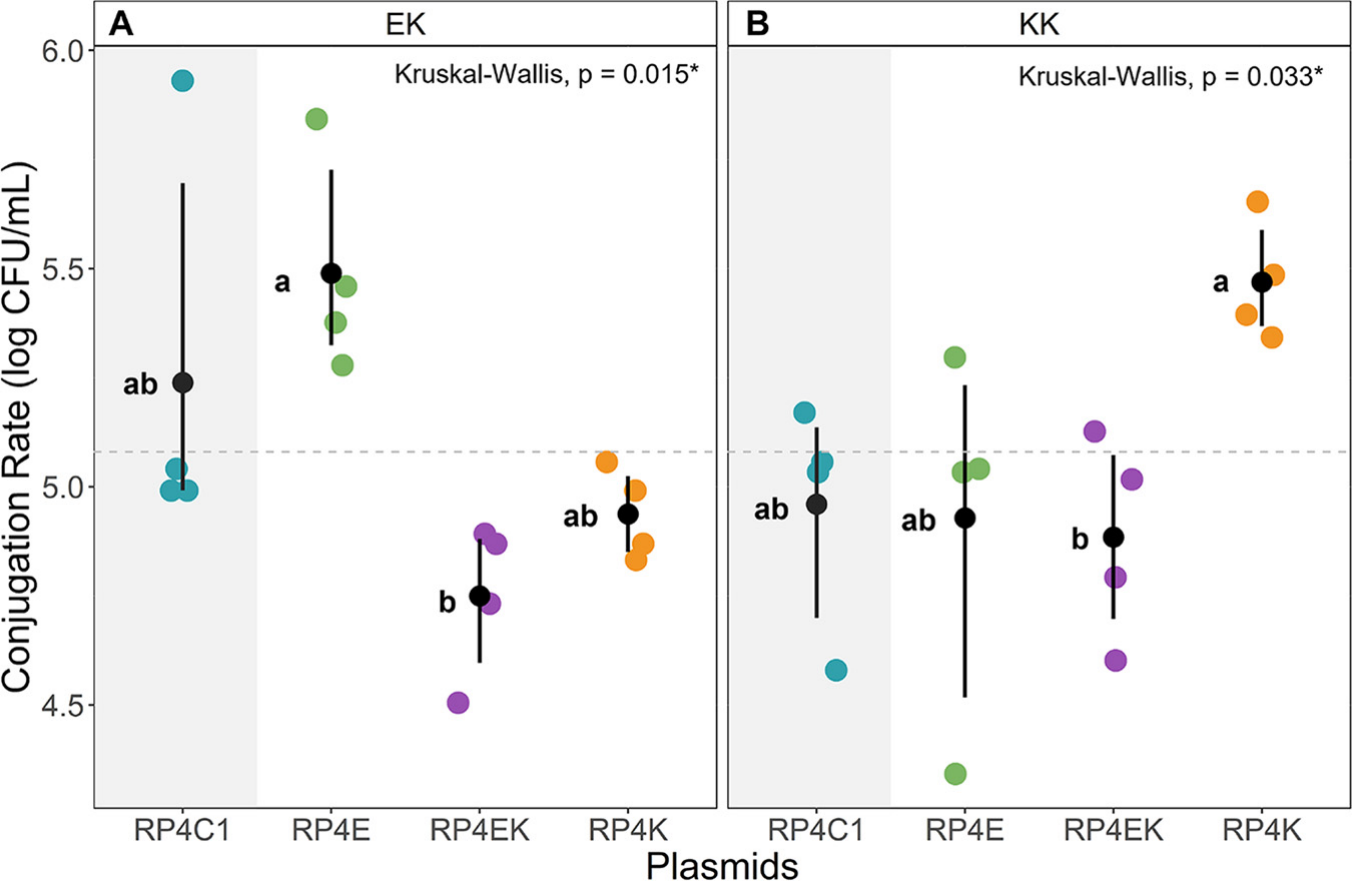
Conjugation rate of evolved RP4 plasmids (RP4E; green, RP4EK; purple, RP4K; orange) and the unevolved RP4 (RP4C1; blue) from biofilm to susceptible planktonic bacteria in: (A). E. coli to K. pneumoniae (EK), and (B). K. pneumoniae to K. pneumoniae (KK) setups. Transfer potential was measured as the conjugation rate (CFU/mL) of each plasmid (N = 4) in the setup. The mean and bootstrap confidence interval of each plasmid can be seen represented by point ranges. A Kruskal-Wallis with Dunn test for post hoc was performed between all plasmid-carrying strains. The P-value is shown, and the letters (a-e) next to each point range indicate whether there is a statistical difference between the plasmids found in the Dunn test. The mean of the entire data can be seen represented in the dashed line.

We studied the effects of past host-history on the conjugation rates from biofilm to planktonic K. pneumoniae in a similar setup as above except the antibiotic concentration was not lethal for the recipient. The E. coli evolved plasmid (RP4E) had a higher mean conjugation rate from E. coli to K. pneumoniae (EK) in comparison to plasmids that evolved entirely or partly with K. pneumoniae (RP4K, RP4EK) (Kruskal-Wallis; Dunn, *P* = 0.015) (Fig. 4A). In the interspecific conjugation setup from E. coli to K. pneumoniae (EK), the interspecifically evolved plasmid (RP4EK) had the lowest conjugation rate. In the intraspecific K. pneumoniae to K. pneumoniae (KK) conjugation setup, the K. pneumoniae evolved plasmid (RP4K) had the highest conjugation rate (Fig. 4B). This indicates that evolution solely in K. pneumoniae improved the within-species horizontal transfer of the plasmid. Interestingly, however, the lowest conjugation rate was seen with the interspecifically evolved plasmid (RP4EK) (Kruskal-Wallis; Dunn, *P* = 0.033) (Fig. 4B). In the KK setup, the RP4C1 and RP4E plasmids had decreased conjugation rate compared to the EK setup, supporting host specificity of the RP4K plasmid. In both of these setups (KK and EK), the K. pneumoniae evolved plasmid (RP4K) confers a higher mean conjugation rate compared to the interspecifically evolved plasmid (RP4EK). However, this was not seen in the evolutionary rescue setups, where RP4K and RP4EK plasmids showed similar results.

### Significant evolution in K. pneumoniae coevolved plasmids generates phenotypic effects.

**FIG 5:**
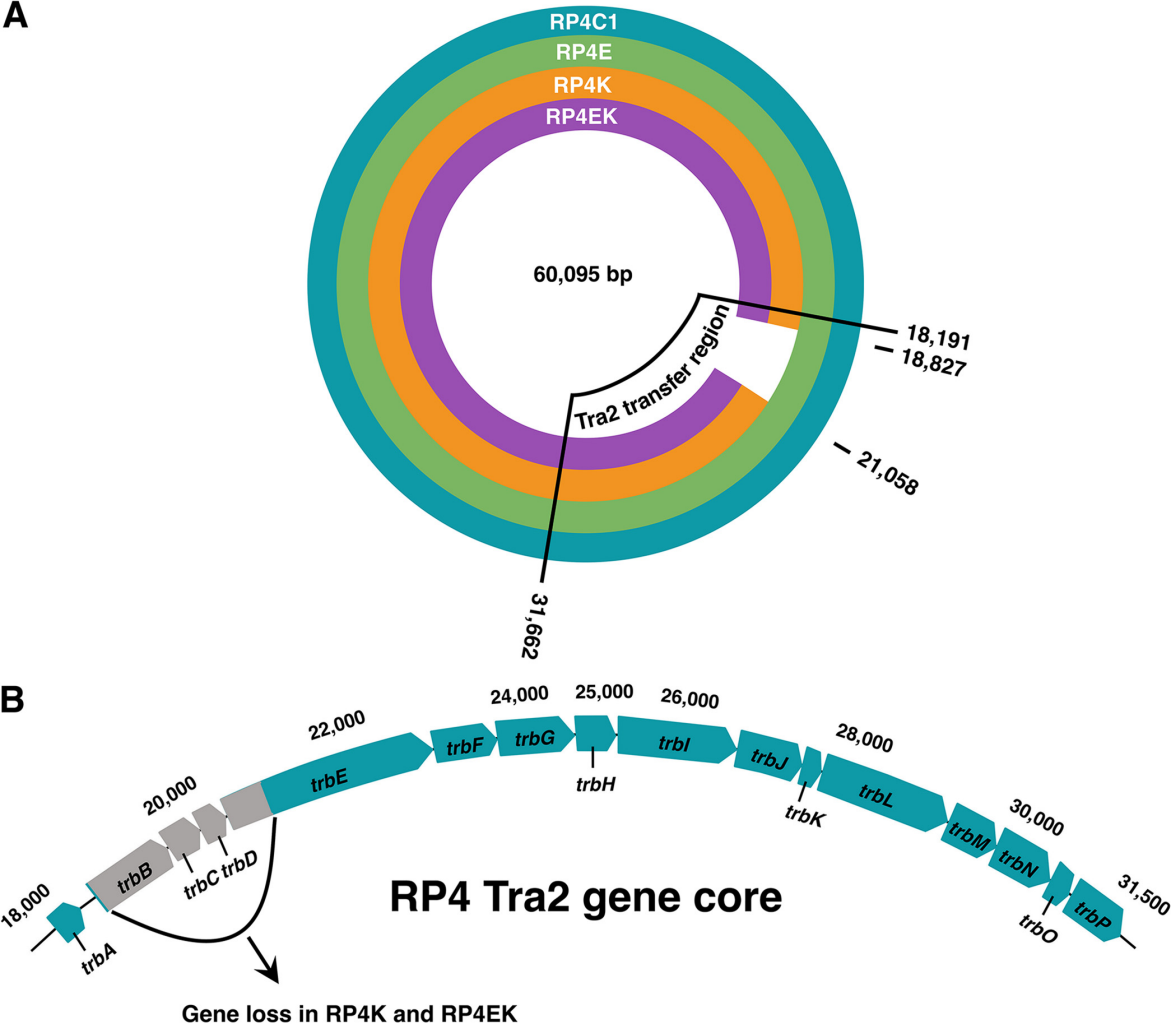
Comparison of the evolved RP4 plasmid sequences. (A) Sequences of RP4 evolved intraspecifically with E. coli (RP4E; green) or K. pneumoniae (RP4K; orange), or interspecifically with them both (RP4EK; purple) were compared with unevolved plasmid (RP4C1; blue). The Tra2 transfer region responsible for the mating-pair formation is highlighted. (B) The genetic organization of Tra2 region with the deletion detected in evolved plasmids RP4K and RP4EK shown in gray.

We sequenced the evolved RP4 plasmids to determine the genetic changes that may confer the phenotypic properties that were observed in plasmids with different host history. We found a major 2,232 bp deletion (with zero coverage in plasmid location 18,827-21,058) located in the Tra2 conjugal transfer region in RP4K and RP4EK plasmids, that were coevolved within setups involving K. pneumoniae (Fig. 5). This deletion affected four genes of the Tra2 complex, *trbB* (960 bp), *trbC* (438 bp), *trbD* (312 bp), and *trbE* (2,559 bp). Almost complete deletion (3-960 bp) was seen for gene *trbB*, complete deletions of *trbC* and *trbD*, and *trbE*, the largest gene of the complex, had a partial deletion of the first 514 bp. Through previous studies, the Tra2 region of RP4 is well established in its role of PRD1 phage propagation (44–49). To test whether the deletion in this region in the RP4K and RP4EK plasmids affects the infectivity of PRD1, we performed spot tests. Bacteria carrying the evolved RP4 plasmids (RP4E, RP4K, and RP4EK) or the unevolved RP4 (RP4C1) as a control were all tested for the susceptibility to PRD1 phage. We observed that while PRD1 was highly infective against bacteria carrying RP4C1 and RP4E plasmids, the bacteria harboring RP4K and RP4EK, that had the partial deletion of the Tra2 transfer region, were found to be immune to this plasmid-dependent phage (Table S2).

10.1128/msphere.00107-23.5 TABLE S2 Drop plaque assay results involving phage PRD1 and the evolved RP4 plasmid as well as unevolved RP4C1 in the bacterial host E. coli HMS174rifR. Download Table S2, PDF file, 0.01 MB. Copyright © 2023 Jonsdottir et al. 2023 Jonsdottir et al. https://creativecommons.org/licenses/by/4.0/ This content is distributed under the terms of the Creative Commons Attribution 4.0 International license.

## DISCUSSION

The usage of antibiotics along with its resistance is on the rise (50). This is because through consumption of antibiotics, resistance is selected for if it is present in the community. Through numerous studies on HGT, in particular via conjugative plasmids, and how it plays in the spread of AMR, its role is well established (3, 51–55). In our study, we investigated a phenomenon known as evolutionary rescue via HGT, which in this case would rely on AMR plasmids being present in the community and spreading during antibiotic treatment to save susceptible bacteria from extinction. This process is associated with certain antibiotics such as beta-lactams and resistance genes that encode degrading enzymes like beta-lactamases or even extended-spectrum beta-lactamases (ESBLs). This is due to the mechanism of the antibiotics which continues to allow for conjugation while the cell is still viable, as well as the resistance mechanism in the case of resistant individuals being present in the community as they lower the antibiotic concentration in their proximity through degradation (36, 37). The evolutionary rescue and conjugation setups performed in this study involved coevolved plasmids and biofilms, which are common bacterial habitats found in the human body. However, we acknowledge that the *in vitro* conditions of this work (LB media, laboratory strains and plasmids) possess limitations on the clinical relevancy of our findings.

In the initial biofilm evolutionary rescue experiment, we investigated the effects of different plasmid characteristics on the rescue potential of the plasmids. There was a significant distinction in the higher rescue potential of the RP4 plasmid compared to the other pEC plasmids. Certain pEC plasmids, pEC15 and pEC14, had little to no rescue potential, which is consistent with the previous studies using these plasmids (36, 56). Little correlation could be drawn between the rescue potential and certain plasmid characteristics such as Inc type, mobility class, and mating pair formation systems. All of these plasmid characteristics were seen to be shared between RP4 and at least one of the pEC plasmids. In natural environments, plasmids are likely to come into contact with each other and interact within cells. The future studies on evolutionary rescue could benefit from involving setups with multiple plasmids which would allow within-host plasmid interactions and might bring differing results than what was presented here. Further, given the different AMR genes encoding various beta-lactamases for each plasmid in this experiment, it is pertinent to investigate whether the rescue potential is affected by the enzyme efficiency.

The different evolutionary histories we created in the RP4 plasmid were tested in the biofilm to planktonic rescue and conjugation setups. It was anticipated that the intraspecifically evolved plasmids (RP4E and RP4K) would have host specificity inferring increased rescue potential or conjugation rate in the intraspecific setup involving their host. Additionally, we were interested in examining the rescue potential or conjugation rate of the interspecifically evolved plasmid (RP4EK) in the interspecific setups. Previous research showed that intraspecific evolutionary history led to host-specialist while interspecific host-plasmid coevolution could lead to host-generalist (57). The interspecific KE rescue setup clearly showed poor plasmid transfer from K. pneumoniae to E. coli. This is consistent with previous results in planktonic setups (23). Throughout our biofilm setups, the E. coli evolved plasmid (RP4E) gave consistent results with the unevolved plasmid (RP4C1). Therefore, it appears reasonable to expect that there was little to no evolutionary influence from the E. coli host during the coevolution. Overall, the K. pneumoniae evolved plasmid (RP4K) and the interspecifically evolved plasmid (RP4EK) grouped together in their relatively low rescue potential and conjugation rate. This grouping seems to indicate a strong evolutionary influence of K. pneumoniae during the coevolution. However, this does not demonstrate RP4EK as a host-generalist with increased rescue potential toward a broader spectrum of hosts. Based on earlier studies involving host switching lineages which might promote adaptation to a new unfavorable host, RP4EK should be tested further on its potential to rescue with an unfamiliar host (43).

Analyzing the growth curves, growth rate, and maximum yield of each evolved plasmid with their coevolved host clearly shows the similarities of RP4K and RP4EK and the distinction of RP4E (Fig. S1, Fig. S2, and Fig. S3). Following this trajectory, the change in rescue potential of the RP4K and RP4EK plasmids was observed in comparison to the unevolved plasmid RP4C1 in the EE setup. Additionally, when examining the significant differences in the rescue potential of RP4EK and the E. coli evolved plasmid RP4E, in the EE rescue, provided that RP4EK was coevolved equally with E. coli and K. pneumoniae. The results seen in the EE rescue setup, indicating clear evolutionary variance distinguishing RP4E and RP4C1 to RP4K and RP4EK was supported by the conventional planktonic conjugation assay (Table S1).

10.1128/msphere.00107-23.1 FIG S1 Evolution of RP4E plasmids coevolved with E. coli host JM109(pSU19). Growth curves were measured as optical density (OD600) in 5 min-intervals of JM109(pSU19) harboring evolved (RP4EC30A-D) or unevolved RP4 (RP4EC1) and plasmid-free bacteria to determine the growth rate (r) and maximum yield (K). Download FIG S1, TIF file, 3.3 MB. Copyright © 2023 Jonsdottir et al. 2023 Jonsdottir et al. https://creativecommons.org/licenses/by/4.0/ This content is distributed under the terms of the Creative Commons Attribution 4.0 International license.

10.1128/msphere.00107-23.2 FIG S2 Evolution of RP4K plasmids evolved with K. pneumoniae host DSM681. Growth curves were measured as optical density (OD_600_) in 5 min-intervals of DSM681 harboring evolved (RP4KC30A-D) or unevolved RP4 (RP4KC1) and plasmid-free bacteria to determine the growth rate (r) and maximum yield (K). Download FIG S2, TIF file, 2.9 MB. Copyright © 2023 Jonsdottir et al. 2023 Jonsdottir et al. https://creativecommons.org/licenses/by/4.0/ This content is distributed under the terms of the Creative Commons Attribution 4.0 International license.

10.1128/msphere.00107-23.3 FIG S3 Evolution of RP4EK plasmids evolved with E. coli host JM109(pSU19) and K. pneumoniae host DSM681. Growth curves were measured as optical density (OD600) in 5 min-intervals of DSM681 harboring evolved (RP4EKC30A-D) or unevolved RP4 (RP4EKC1) and plasmid-free bacteria to determine the growth rate (r) and maximum yield (K). Download FIG S3, TIF file, 3.6 MB. Copyright © 2023 Jonsdottir et al. 2023 Jonsdottir et al. https://creativecommons.org/licenses/by/4.0/ This content is distributed under the terms of the Creative Commons Attribution 4.0 International license.

10.1128/msphere.00107-23.4 TABLE S1 Mean conjugation frequency per donor cell from the conjugation assay replicating the setup of the biofilm experiments (N = 4). Download Table S1, PDF file, 0.01 MB. Copyright © 2023 Jonsdottir et al. 2023 Jonsdottir et al. https://creativecommons.org/licenses/by/4.0/ This content is distributed under the terms of the Creative Commons Attribution 4.0 International license.

A reasonable explanation for the close results of RP4K and RP4EK is the identical deletion found in both of their sequences, localized in the Tra2 core complex, responsible for mating pair formation in the RP4 plasmid. This deletion was not seen with the RP4E plasmid, indicating less adaptation involving E. coli host lineage. This is contrary to previous research where E. coli host lineage caused major deletions in a plasmid, including the conjugative machinery, while evolutionary history with K. pneumoniae brought no major genetic changes (58). Three genes were practically completely deleted, *trbB*, *trbC*, and *trbD* and a fourth gene *trbE* had a partial deletion. Previous studies have reported that these genes are both essential for the conjugal transfer as well as phage propagation for the plasmid-dependent phage PRD1 (44–49, 59). This study clearly shows continued conjugal transfer in all setups although decreased for RP4K and RP4EK plasmids presumably due to the lack of mating pair formation genes that were previously noted to be essential (45, 59). This may suggest that biofilms better preserve the transfer of the RP4 plasmid even in the absence of seemingly essential conjugal transfer genes as the previous studies tested conjugation in liquid planktonic conjugation setups (45, 59). In this study, the host-plasmid coevolution was performed in liquid planktonic cultures. Although there was a constant antibiotic selection for the plasmid, the host-plasmid coevolution could have provided alternative results had they been performed in biofilm, as it may support the preservation of the conjugal transfer genes that were lost in RP4K and RP4EK. This is also supported by a recent study that found that plasmid persistence in planktonic communities was improved by loss of conjugal transfer genes, compared to biofilm communities in which the genes were retained (60). It seems that deletions of the conjugal transfer region are common when the plasmid confers a high cost to the host. This does not seem to be linked to a specific species, but rather how favorable the host-plasmid pairings are (58). In our study, K. pneumoniae could have selective pressure for alleviating the cost of the plasmid and confer fitness advantages through the deletion of the costly genes.

Interestingly, one difference from the grouping pattern of RP4K and RP4EK was observed. In the KK conjugation setup, the RP4K plasmid clearly inferred a higher rescue potential compared to the other plasmids, suggesting increased host specificity. This host specificity seemingly due to the intraspecific coevolution is what we had anticipated. However, this strays away from the genotypic-to-phenotypic patterns for the RP4K and RP4EK plasmids, as they have identical sequences and highly similar results in the other setups. The RP4K plasmid in the KK conjugation setup provides a better conjugation rate that cannot be explained by the plasmid sequence. This could be due to an unknown interaction between the evolved plasmid and host, perhaps epigenetic modifications. As we mentioned above, the deleted genes in RP4K and RP4EK had previously been described as essential in conjugal transfer and for plasmid-dependent-phage PRD1 propagation. Through a simple spot test assay, it was clear that strains carrying the evolved plasmids RP4K and RP4EK inferred immunity to the PRD1 phage. This supports the previous research on the Tra2 core and its essential role in the phage propagation for PRD1 (44). Along with the conceivable fitness advantages in the coevolved planktonic community, the loss of the conjugal transfer genes of RP4K and RP4EK could serve as an exaptation that provide significant advantage in the presence of a phage.

Our findings demonstrate that evolutionary rescue via conjugative plasmids is possible in a biofilm to planktonic setup, even with the lack of conjugal transfer genes. We show here that even relatively short periods of history in specific host can have a significant effect on plasmid’s rescue potential and conjugation rate. The hosts used in this study, E. coli and K. pneumoniae, clearly exhibit different evolutionary influence on the RP4 plasmid, although the hosts are relatively similar. As such, it is possible that plasmid populations are continuously balancing between the benefits and costs of intra- and interspecific adaptations. These adaptations may determine the plasmid’s survival in highly adverse conditions (for their hosts) such as in the sudden presence of lethal antibiotic doses and lytic bacteriophages.

## MATERIALS AND METHODS

### Bacterial strains and plasmids.

The bacterial plasmid hosts used in this study were strains of two species of *Enterobacteriaceae*, Escherichia coli and Klebsiella pneumoniae. The plasmids used in this study were RP4, a broad-host-range conjugative plasmid that has a high conjugation rate (61–64), and five ESBL-plasmids isolated from clinical E. coli strains (pEC plasmids; [36]) (Table 1). Lysogeny broth (LB) (65), supplemented with 1% agar and/or antibiotics as indicated, were used for bacterial cultivation. Bacterial cultures were grown at 37°C with 200 rpm agitation unless otherwise specified and on agar plates incubated at 37°C.

**TABLE 1:** The bacterial strains and plasmids used in this study

Strain	Plasmid(s)	Plasmid size (bp)	Inc type	MPF^*d*^ type	MOB^*d*^ class	β-lactamase identified	Other resistance genes
E. coli JM109 (pSU19)*^CamR^*	pSU19^*a*^	2340	-	-	-	-	*cat*
E. coli HMS174 (plasmid-free)*^RifR^*	-	-	-	-	-	-	-
E. coli HMS174 (pEC3)*^RifR^*^,^*^AmpR^*	pEC3pl1 pEC3pl2	91,885 59,192 (59,192)^*c*^	IncB/ O/ K/ Z IncI2	MPFI MPFT	MOBP MOBP	*blaTEM-1C* *-*	*strA, strB, sul2* -
E. coli HMS174 (pEC13)*^RifR^*^,^*^AmpR^*	pEC13	71,656	IncFII	MPFF	MOBF	*blaCTX-M-14*	-
E. coli HMS174 (pEC14)*^RifR^*^,^*^AmpR^*	pEC14pl1 pEC14pl2	143,590 87,848 (87,666)^*c*^	IncFII, IncQ1, IncP, IncFIB IncI1	MPFF MPFI	MOBF MOBP	*blaTEM-1B* -	*strA,strB, aadA1, mph(B), sul1, sul2, tet(A), dfrA1* -
E. coli HMS174 (pEC15)*^RifR^*^,^*^AmpR^*	pEC15pl1 pEC15pl2	87,811 (87,767)^*c*^ 38,611	IncI1 IncX1	MPFI MPFT	MOBP MOBQ	- *blaTEM-52B*	- -
E. coli HMS174 (pEC16)*^RifR^*^,^*^AmpR^*	pEC16pl1 pEC16pl2^*b*^	94,325 (95,380)^*c*^ 7,939	IncI1 ColRNAI	MPFF -	MOBP MOBP	*blaSHV-12* *-*	- -
E. coli JM109(pSU19) (RP4)*^CamR, AmpR, KanR, TetR^*	pSU19^*a*^ RP4	60,095	IncP-1α	MPFT	MOBP	*blaTEM−2*	*cat* *tet(A), aph(3′)-lb*
E. coli HMS174(RP4)*^RifR, AmpR, KanR, TetR^*	RP4	60,095	IncP-1α	MPFT	MOBP	*blaTEM-2*	*tet(A), aph(3′)-lb*
K. pneumoniae DSM681 (plasmid-free)*^RifR, AmpR^*	-	-	-	-	-	*blaSHV-28*	*-*
K. pneumoniae DSM681 (plasmid-free)*^StrepR, AmpR^*	-	-	-	-	-	*blaSHV-28*	*-*
K. pneumoniae DSM681(RP4)*^RifR, AmpR, KanR, TetR^*	RP4	60,095	IncP-1α	MPFT	MOBP	*blaTEM-2, blaSHV-28*	*tet(A), aph(3′)-lb*
K. pneumoniae DSM681(RP4)*^StrepR, AmpR, KanR, TetR^*	RP4	60,095	IncP-1α	MPFT	MOBP	*blaTEM-2, blaSHV-28*	*tet(A), aph(3′)-lb*

a Nonconjugative plasmid.

b Nonconjugative mobilizable plasmid.

c Alterations to plasmid size due to the shufflon area are indicated in parentheses.

^*d*^MPF = mating pair formation; MOB = mobility.

### Host-plasmid coevolution experiments.

The purpose of the experiments was to evolve the RP4 plasmids to intra- and interspecific host systems involving E. coli and/or K. pneumoniae. The naming of each treatment was the RP4 plasmid indicated by the first letter of the genus name of the host strains used (RP4E, RP4K, RP4EK). The host-plasmid coevolution treatments were initiated in 5 mL LB broth with host bacterial strains containing the RP4 plasmid and carried out for 30 cycles. The bacterial hosts were JM109(pSU19) harboring plasmid pSU19 encoding chloramphenicol resistance (camR) and DSM681 with chromosomal mutations encoding rifampicin resistance (rifR) (66, 67). For each cycle, the culture transfers were done at 1:1000 dilution with appropriate antibiotic selection to select for host and plasmid. For treatment RP4E, the medium was supplemented with 25 μg/mL chloramphenicol and 25 μg/mL kanamycin, and for treatment RP4K with 150 μg/mL rifampicin and 25 μg/mL kanamycin. For treatment RP4EK, each host was resistant to a separate antibiotic, this allowed host antibiotics to be swapped sequentially to encourage plasmid transfer between the two host species, allowing one host strain to maintain the plasmid at once. The antibiotic selection for treatment RP4EK involved the following five cycle supplementation that was repeated six times for a total of 30 cycles: (I) 25 μg/mL kanamycin, (II) 15 μg/mL rifampicin and 2.5 μg/mL kanamycin, (III) 150 μg/mL rifampicin and 25 μg/mL kanamycin, (IV) 2.5 μg/mL chloramphenicol and 2.5 μg/mL kanamycin, (V) 25 μg/mL chloramphenicol and 25 μg/mL kanamycin. This experiment was run with four biological replicates per population. Growth analysis was done to compare the starting point (cycle 1) to the endpoint (cycle 30) of each population, as well as with the plasmid-free host. Overnight cultures were initiated in LB broth (with 25 μg/mL kanamycin for RP4 plasmid carrying strains) before 1:100 dilution was performed into fresh LB medium and mixed thoroughly. The growth of the bacterial strains was determined with a Bioscreen C MBR machine (Bioscreen, Oy Growth Curves Ab Ltd.) for 24 h as described previously (56). The growth curves, growth rate (r), and maximum yield (K) were calculated from the data using RStudio (R version 4.2.1), with R source code based on a previously described MATLAB code (68).

### Biofilm experiments: rescue potential and conjugation efficiency.

The evolutionary rescue potential of the plasmids was studied by the capacity of plasmid-carrying biofilm to rescue the planktonic antibiotic-susceptible bacteria. The setup involved the evolved RP4 plasmids (RP4E, RP4K, and RP4EK) and unevolved RP4C1, from cycle 1, tested under two rescue setups through a combination of two donor strains, E. coli HMS174^rifR^ and K. pneumoniae DSM681^rifR^, and the susceptible recipient strain E. coli JM109(pSU19)^camR^. The setups were given abbreviations indicating the donor and recipient strains, respectively (EE and KE; E for E. coli and K for K. pneumoniae). Additionally, five previously characterized ESBL-conferring plasmids (pEC3, pEC13-16) were tested in the EE rescue setup. The conjugation efficiency of the evolved RP4 plasmids from biofilm to planktonic cells was tested in setups EK and KK, with the same donor strains as the rescue setups and the nonsusceptible K. pneumoniae DSM681^strepR^ as the recipient strain.

The biofilm experiments were started by inoculating 25 μL of the overnight donor cultures, grown in LB supplemented with 150 μg/mL ampicillin, into fresh 5 mL LB with 150 μg/mL ampicillin and 180 μL were aliquoted into wells of a 96-well plate (Nunc MicroWell, Thermo Scientific) in 4 replicates/culture. Plasmid-free strains (E. coli HMS174^rifR^, K. pneumoniae DSM681^rifR^) were used as a control. The plate was closed with a 96-pin lid (Nunc Immuno TSP Lid, Thermo Scientific), sealed with parafilm, to allow biofilm to grow onto the pins for 5 days at 37°C without shaking. After the 5-day incubation, the lid with the biofilm-covered pins was washed two times with 1× PBS before being introduced to a new 96-well plate containing 180 μL planktonic recipient strain. The recipient strain had been grown overnight, before being transferred at 1:1000 dilution into fresh media supplemented with 150 μg/mL ampicillin for 1 h. The length of antibiotic exposure before rescue and type of antibiotics used were based on previous research (36, 37). The plate was sealed with parafilm and grown overnight without shaking. Dilutions of the product of each well were plated on LB agar plates with appropriate antibiotics selecting for only the recipient and plasmid to determine the density of the formed transconjugants as CFU (CFU). Rescue setup EE selected for transconjugants with 25 μg/mL chloramphenicol and 150 μg/mL ampicillin, while setup KE selected with 25 μg/mL chloramphenicol and 25 μg/mL kanamycin. Conjugation setups EK and KK selected for transconjugants with 25 μg/mL streptomycin and 25 μg/mL kanamycin.

### Conjugation assay.

Conventional planktonic conjugation assay with the evolved and unevolved RP4 plasmids were performed alongside the biofilm experiments with the same donors and recipients. This was done to measure the mean conjugation frequency (CFU/mL) per donor cell and compare with the biofilm experiments as they were unable to determine the donor cell density. The conjugation was done by adding 5 μL of the plasmid-carrying donor overnight culture and 500 μL of the recipient overnight culture in 5 mL LB for 2 h at 37°C, 200 rpm. The product of the conjugation was then plated on LB agar with appropriate antibiotics to determine the cell density (CFU/mL) of the formed transconjugants. Transconjugants for conjugation assays EE and KE were selected for with 25 μg/mL chloramphenicol and 25 μg/mL kanamycin, and for conjugation assays EK and KK, transconjugants were selected with 25 μg/mL streptomycin and 25 μg/mL kanamycin. The donor strains were plated on LB agar supplemented with 150 μg/mL rifampicin to determine their cell number (CFU). The mean conjugation frequency was given as the transconjugant cell density divided by donor cell number.

### Plaque assay.

To determine the infectivity of the PRD1 phage, which is dependent on the RP4 mating pair formation complex, spot test plaque assays were performed (69). The plaque assays were initiated by combining 3 mL of melted LB soft-agar (0.7%) with 100 μL of overnight grown plasmid-carrying host and then poured onto LB agar plates. PRD1 viral lysate (6.9 × 10^10^ PFU/mL; plaque forming units) was then spotted (10 μL) onto the plates. The plates were grown overnight at 37°C.

### Plasmid sequencing and bioinformatic analyses.

To explore the possible mutations in the evolved RP4 plasmids, the total DNA from clonal populations of the E. coli HMS174^rifR^ strain carrying RP4E, RP4K, RP4EK, and RP4C1 (as reference) was isolated using Wizard genomic DNA purification kit (Promega) according to the manufacturer’s instructions. The DNA concentration was determined with a Qubit 3.0 fluorometer using the dsDNA HS kit (Invitrogen, ThermoFisher Scientific). The sequencing library preparation was done with NEB Next Ultra DNA Library Prep kit and 2 × 150 bp paired-end (PE150) DNA sequencing was performed on Illumina NovaSeq 6000 platform with S4 flowcell for the strains carrying the evolved plasmids. The illumina reads were trimmed with trimmomatic (0.39) (70). Trimmomatic was run as paired end mode to trim for illumina adapters (ILLUMINACLIP with the following settings: 2 for seed mismatches; 30 for palindrome clip threshold; 10 for simple clip threshold; and 2 as minimum adapter length in palindrome mode in keepBothReads setting) and for quality (SLIDINGWINDOW with 3 for window size and 21 for average quality threshold). Reads with length under 100 bp after trimming were discarded from the analysis.

RP4C1 served as an unevolved control and was used for short-insert library preparation and sequenced with DNBSEQ platform (PE150). The reads were quality- and adapter-trimmed with SOAPnuke (71) by the sequencing service; reads containing more than 1% of N, more than 40% of the bases in a read have quality value under 20, or reads with length under 150 bp were removed. The corrected reads were mapped to reference RP4 sequence (BN000925.1) to detect possible genetic changes using the mutation prediction pipeline breseq (0.37.0) with consensus mode to detect mutations that exist in the clonal samples at 100% frequency (72). The mutations detected in RP4C1 were filtered out from the evolved plasmid mutations manually. The overall coverages were 435 (RP4C1), 1127 (RP4E), 471 (RP4K) and 522 (RP4EK). The sequencing coverage of the deletion site was 477 for RP4K and 547 for RP4EK. The Geneious Prime software version 2022.2.2 (Geneious) was used to further visualize specific mutations in the mapped plasmid sequences.

### Statistical analysis.

All statistical analysis was carried out in RStudio (R version 4.2.1) (see code in supplemental material, Supplemental Text File S1). The dependent variable (CFU/mL) was transformed on a log scale. The possible statistical significance of each plasmid in each rescue and conjugation setup was determined through either a one-way ANOVA with Tukey-HSD as *post hoc* comparisons or a Kruskal-Wallis with Dunn test as *post hoc* comparisons. The statistical significance between plasmids found with the Tukey and Dunn tests was indicated through a compact letter display on the figures.

10.1128/msphere.00107-23.6 TEXT S1 R code for statistical analysis of evolutionary rescue and conjugation setup results. Download Text S1, PDF file, 0.05 MB. Copyright © 2023 Jonsdottir et al. 2023 Jonsdottir et al. https://creativecommons.org/licenses/by/4.0/ This content is distributed under the terms of the Creative Commons Attribution 4.0 International license.
