# Supplementary material for: Preceding Host History of Conjugative Resistance Plasmids Affects Intra- and Interspecific Transfer Potential from Biofilm
Source: mSphere. 2023 Apr 5;8(3):e00107-23. doi: 10.1128/msphere.00107-23 (PMC10286713; doi:10.1128/msphere.00107-23)
Supplement: TABLE S2 [file msphere.00107-23-s0006.pdf]

Plasmid	PRD1 plaques (viral lysate drop: 6.9×10^8 pfu/10 µL)
RP4C1	Plaque formed
RP4E	Plaque formed
RP4K	No plaque
RP4EK	No plaque
