# Supplementary figures and images for: Preceding Host History of Conjugative Resistance Plasmids Affects Intra- and Interspecific Transfer Potential from Biofilm
Source: mSphere. 2023 Apr 5;8(3):e00107-23. doi: 10.1128/msphere.00107-23 (PMC10286713; doi:10.1128/msphere.00107-23)

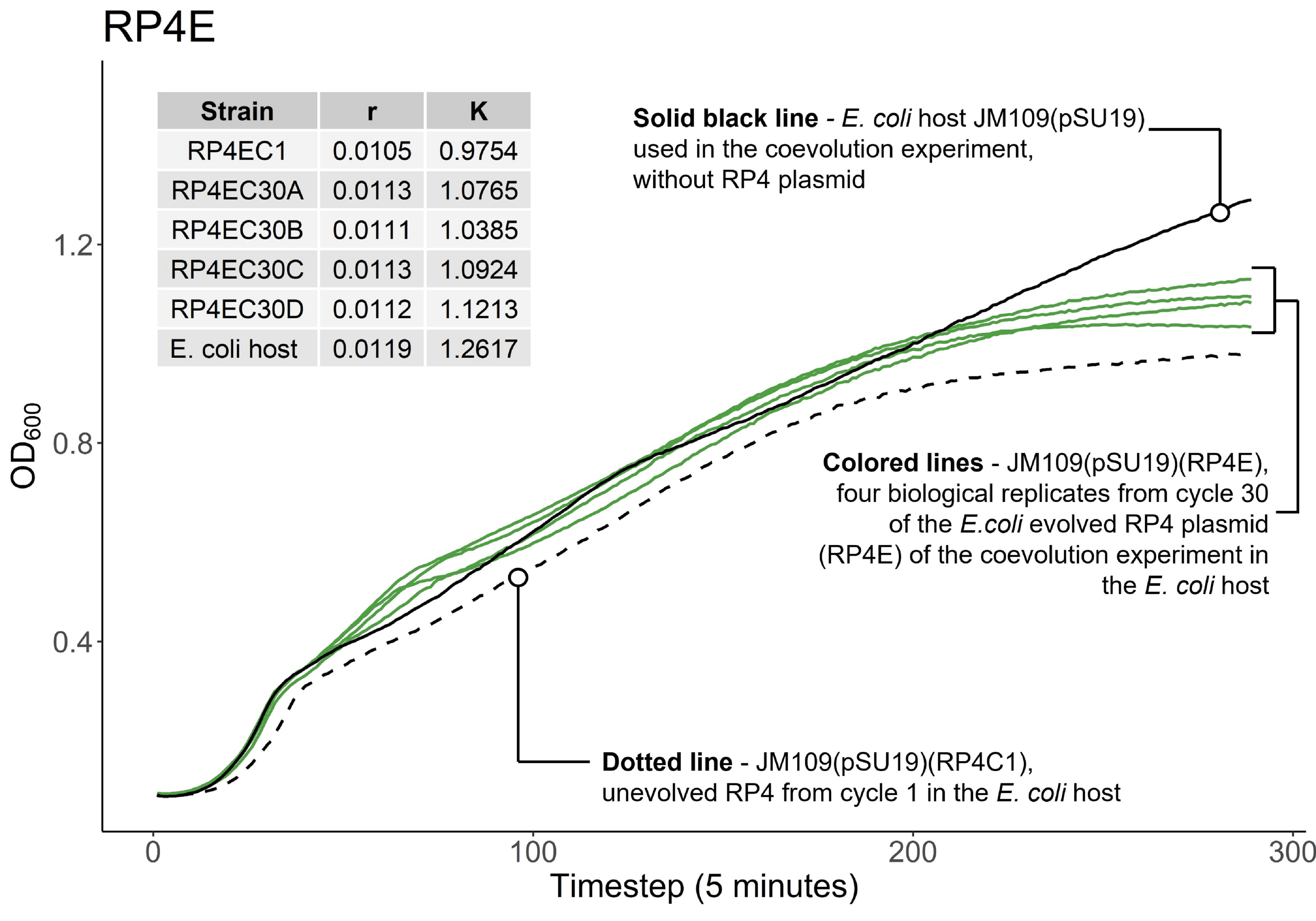

Supplement: FIG S1 [file msphere.00107-23-s0001.tif]

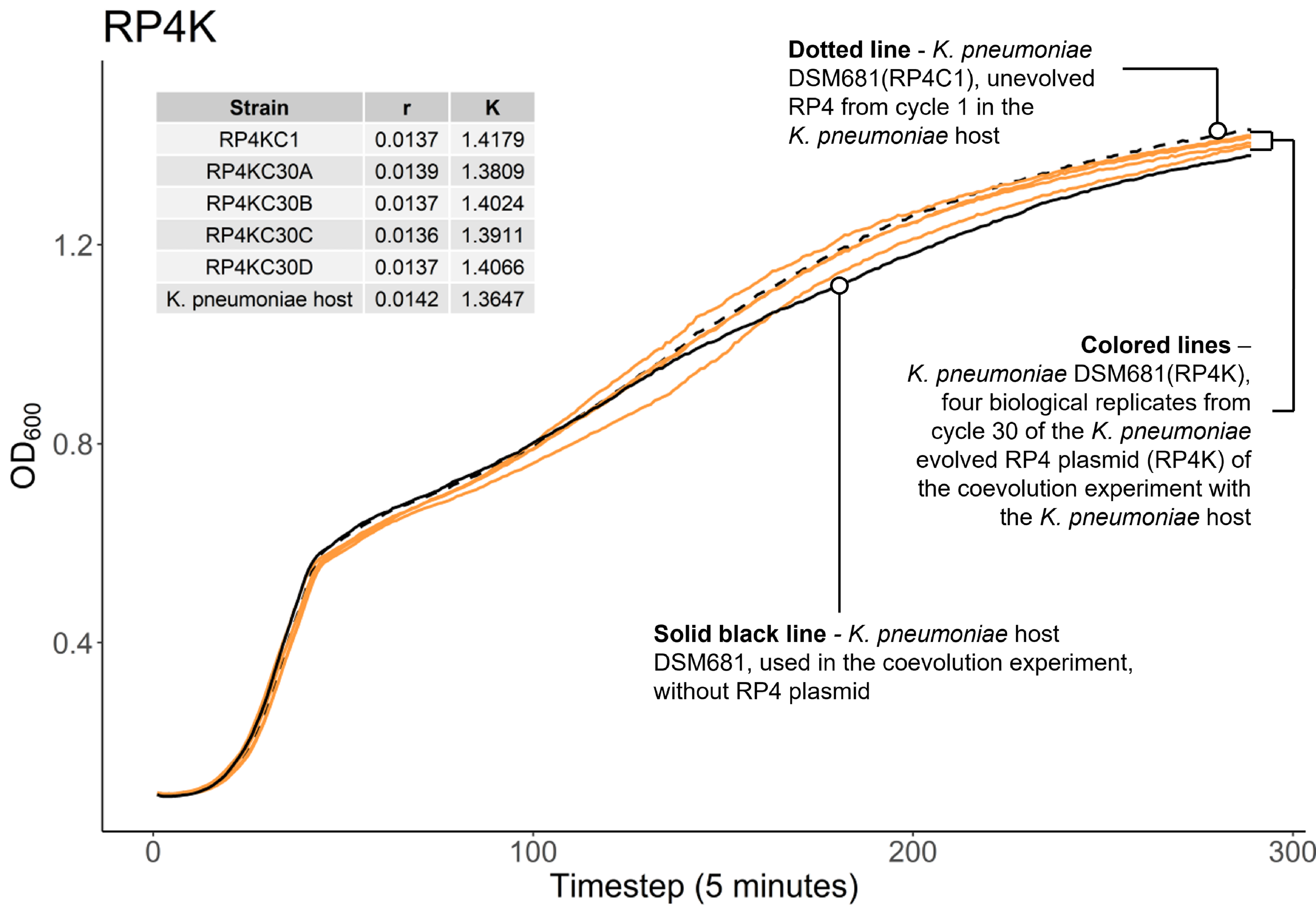

Supplement: FIG S2 [file msphere.00107-23-s0002.tif]

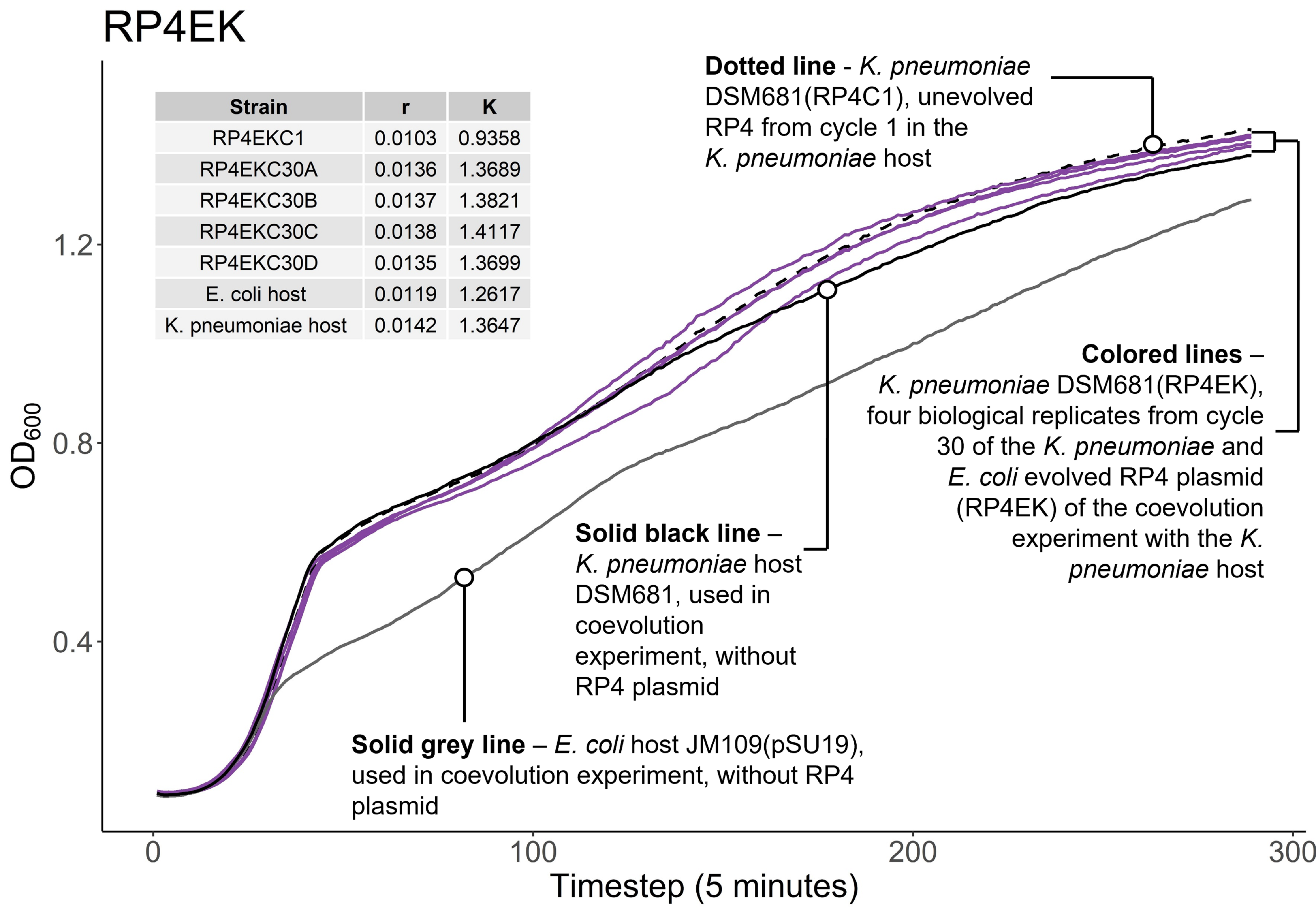

Supplement: FIG S3 [file msphere.00107-23-s0003.tif]
