# Supplementary material for: Preceding Host History of Conjugative Resistance Plasmids Affects Intra- and Interspecific Transfer Potential from Biofilm
Source: mSphere. 2023 Apr 5;8(3):e00107-23. doi: 10.1128/msphere.00107-23 (PMC10286713; doi:10.1128/msphere.00107-23)
Supplement: TABLE S1 [file msphere.00107-23-s0005.pdf]

Conjugation System	Plasmids			
	RP4C1	RP4E	RP4K	RP4EK
EE	6.533333e-03	1.153333e-02	5.054054e-06	5.714286e-06
EK	3.181481e-05	2.770000e-05	8.933333e-06	6.085714e-06
KE	7.017544e-06	3.361345e-06	1.250000e-05	6.993007e-06
KK	4.351724e-06	4.506494e-06	4.853333e-06	2.879747e-06
