# Supplementary material for: Preceding Host History of Conjugative Resistance Plasmids Affects Intra- and Interspecific Transfer Potential from Biofilm
Source: mSphere. 2023 Apr 5;8(3):e00107-23. doi: 10.1128/msphere.00107-23 (PMC10286713; doi:10.1128/msphere.00107-23)
Supplement: TEXT S1 [file msphere.00107-23-s0004.pdf]

```
################### Statistics EVOLVED RP4 EvoResQ data ####################
#packages
library(multcompView)
library(dplyr)
library(lmtest)
library(car)
library(caret)
library(FSA)

#load in data
dataRP4vpEC <- read.csv("evoResQ_pECvRP4.csv", sep="\t", dec = ",", header=TRUE)
dataRP4vpEC

dataEE <- read.csv("evolvedRP4_evoResQ_EE.csv", sep="\t", dec = ",", header=TRUE)
dataEE

dataKE <- read.csv("evolvedRP4_evoResQ_KE.csv", sep="\t", dec = ",", header=TRUE)
dataKE

dataEK <- read.csv("evolvedRP4_conj_EK.csv", sep="\t", dec = ",", header=TRUE)
dataEK

############## pEC vs RP4 ###############
#model
lmModRP4vpEC <- lm(dataRP4vpEC$log_CFU_mL ~ dataRP4vpEC$Plasmid,
data=dataRP4vpEC) # initial model

par(mfrow=c(2,2)) # init 4 charts in 1 panel

plot(lmModRP4vpEC)
#BP test
lmtest::bptest(lmModRP4vpEC)  # Breusch-Pagan test
car::ncvTest(lmModRP4vpEC)  # NCV test
```

```
#testing a different way to see if I get consistent results
fitRp <- lm(dataRP4vpEC$log_CFU_mL~dataRP4vpEC$Plasmid)
ncvTest(fitRp)

#view model summary
summary(fitRp)

#residuals
resRp <- resid(fitRp)
resRp
#produce residual vs. fitted plot
plot(fitted(fitRp), resRp)
#add a horizontal line at 0
abline(0,0)
#create Q-Q plot for residuals
qqnorm(resRp)
#add a straight diagonal line to the plot
qqline(resRp)
#Create density plot of residuals
plot(density(resRp))

#check heteroskedasticity
lmcRp <- lm(log_CFU_mL ~ Plasmid, data = dataRP4vpEC)
gvlma(lmcRp) #assumptions met!!!

## ANOVA ##
# analysis of variance for RP4 vs pEC rescue

dataRP4vpEC
anova.dataRp <- aov(log_CFU_mL ~ as.factor(Plasmid), data = dataRP4vpEC)
summary(anova.dataRp)
```

```
# table with factors, means and standard deviation
dataRp_summary <- group_by(dataRP4vpEC, Plasmid) %>%
        summarise(mean=mean(log_CFU_mL), sd=sd(log_CFU_mL)) %>%
        arrange(desc(mean))
print(dataRp_summary)

# Tukey's test
tukey.dataRp <- TukeyHSD(anova.dataRp)
print(tukey.dataRp)

# creating the compact letter display
cld.dataRp <- multcompLetters4(anova.dataRp, tukey.dataRp)
print(cld.dataRp)

# adding the compact letter display to the table with means and sd
cldRp <- as.data.frame.list(cld.dataRp$`as.factor(Plasmid)`)
dataRp_summary$Tukey <- cldRp$Letters
print(dataRp_summary)

############## EE ###############
#model
lmModEE <- lm(dataEE$log_CFU_mL ~ dataEE$Plasmid, data=dataEE) # initial model

par(mfrow=c(2,2)) # init 4 charts in 1 panel

plot(lmModEE)
#BP test
lmtest::bptest(lmModEE)  # Breusch-Pagan test
car::ncvTest(lmModEE)  # NCV test
#testing a different way to see if I get consistent results
fitEE <- lm(dataEE$log_CFU_mL~dataEE$Plasmid)
ncvTest(fitEE)
```

```
#view model summary
summary(fitEE)

#residuals
resEE <- resid(fitEE)
resEE

#produce residual vs. fitted plot
plot(fitted(fitEE), resEE)
#add a horizontal line at 0
abline(0,0)
#create Q-Q plot for residuals
qqnorm(resEE)
#add a straight diagonal line to the plot
qqline(resEE)
#Create density plot of residuals
plot(density(resEE))

#check heteroskedasticity
lmcEE <- lm(log_CFU_mL ~ Plasmid, data = dataEE)
gvlma(lmcEE) #assumptions NOT met!!! will need to do transformation

# square root transformation
sqrt_CFU_mL <- sqrt(dataEE$CFU_mL)

dataEE <- cbind(dataEE, sqrt_CFU_mL)
head(dataEE)

lmcEE_sqrt <- lm(sqrt_CFU_mL ~ Plasmid, data = dataEE)
gvlma(lmcEE_sqrt) # assumptions met!!!
```

```
## ANOVA ##
# analysis of variance in EE rescue
dataEE
anova.dataEE <- aov(sqrt_CFU_mL ~ as.factor(Plasmid), data = dataEE)
summary(anova.dataEE)

# table with factors, means and standard deviation
dataEE_summary <- group_by(dataEE, Plasmid) %>%
    summarise(mean=mean(sqrt_CFU_mL), sd=sd(sqrt_CFU_mL)) %>%
    arrange(desc(mean))
print(dataEE_summary)

# Tukey's test
tukey.dataEE <- TukeyHSD(anova.dataEE)
print(tukey.dataEE)

# creating the compact letter display
cld.dataEE <- multcompLetters4(anova.dataEE, tukey.dataEE)
print(cld.dataEE)

# adding the compact letter display to the table with means and sd
cldEE <- as.data.frame.list(cld.dataEE$`as.factor(Plasmid)`)
dataEE_summary$Tukey <- cldEE$Letters
print(dataEE_summary)

### Kruskal wallis test log transformed ###
kruskalEE <- kruskal.test(log_CFU_mL ~ Plasmid, data = dataEE)
summary(kruskalEE)
kruskalEE

### KW test post hoc dunn test ###
dunnEE <- dunnTest(log_CFU_mL ~ Plasmid,
```

```
          data = dataEE,
          method = "holm")
dunnEE

############## KE ###############
#model
lmModkE <- lm(dataKE$log_CFU_mL ~ dataKE$Plasmid, data=dataEE) # initial model

par(mfrow=c(2,2)) # init 4 charts in 1 panel

plot(lmModKE)
#BP test
lmtest::bptest(lmModKE)  # Breusch-Pagan test
car::ncvTest(lmModKE)  # NCV test
#testing a different way to see if I get consistent results
fitKE <- lm(dataKE$log_CFU_mL~dataKE$Plasmid)
ncvTest(fitKE)

#view model summary
summary(fitKE)

#residuals
resKE <- resid(fitKE)
resKE

#produce residual vs. fitted plot
plot(fitted(fitKE), resKE)
#add a horizontal line at 0
abline(0,0)
#create Q-Q plot for residuals
qqnorm(resKE)
#add a straight diagonal line to the plot
```

```
qqline(resKE)
#Create density plot of residuals
plot(density(resKE))

#check heteroskedasticity
lmcKE <- lm(log_CFU_mL ~ Plasmid, data = dataKE)
gvlma(lmcKE) #assumptions NOT met!!! will need to do transformation

# square root transformation
sqrt_CFU_mL <- sqrt(dataKE$CFU_mL)

dataKE <- cbind(dataKE, sqrt_CFU_mL)
head(dataKE)

lmcKE_sqrt <- lm(sqrt_CFU_mL ~ Plasmid, data = dataKE)
gvlma(lmcKE_sqrt) # assumptions NOT met!!!

# cubic root transformation
cubrt_CFU_mL <- dataKE$CFU_mL^(1/3)

dataKE <- cbind(dataKE, cubrt_CFU_mL)
head(dataKE)

lmcKE_cubrt <- lm(cubrt_CFU_mL ~ Plasmid, data = dataKE)
gvlma(lmcKE_cubrt) # assumptions NOT met!!!

# recip transformation
rec_CFU_mL <- dataKE$CFU_mL^(-1)

dataKE <- cbind(dataKE, rec_CFU_mL)
head(dataKE)
```

```
lmcKE_rec <- lm(rec_CFU_mL ~ Plasmid, data = dataKE)
gvlma(lmcKE_rec) # assumptions NOT met!!!



# BoxCox transformation
BC_CFU_mL <- BoxCoxTrans(dataKE$CFU_mL)

dataKE <- cbind(dataKE, BC_CFU_mL = predict(BC_CFU_mL, dataKE$CFU_mL))
head(dataKE)

BClmcKE <- lm(BC_CFU_mL ~ Plasmid, data = dataKE)
gvlma(BClmcKE) # assumptions NOT met!!!

# sqrt recip transformation
sqrtrec_CFU_mL <- dataKE$sqrt_CFU_mL^(-0.5)

dataKE <- cbind(dataKE, sqrtrec_CFU_mL)
head(dataKE)

lmcKE_sqrtrec <- lm(sqrtrec_CFU_mL ~ Plasmid, data = dataKE)
gvlma(lmcKE_sqrtrec) # assumptions NOT met!!!

## ANOVA ##
# analysis of variance in KE rescue
dataKE
anova.dataKE <- aov(log_CFU_mL ~ as.factor(Plasmid), data = dataKE)
summary(anova.dataKE)

# table with factors, means and standard deviation
dataKE_summary <- group_by(dataKE, Plasmid) %>%
	summarise(mean=mean(log_CFU_mL), sd=sd(log_CFU_mL)) %>%
	arrange(desc(mean))
```

```
print(dataKE_summary)

# Tukey's test
tukey.dataKE <- TukeyHSD(anova.dataKE)
print(tukey.dataKE)

# creating the compact letter display
cld.dataKE <- multcompLetters4(anova.dataKE, tukey.dataKE)
print(cld.dataKE)

# adding the compact letter display to the table with means and sd
cldKE <- as.data.frame.list(cld.dataKE$`as.factor(Plasmid)`)
dataKE_summary$Tukey <- cldKE$Letters
print(dataKE_summary)

#ANOVA non transformed
anova.dataKE <- aov(CFU_mL ~ as.factor(Plasmid), data = dataKE)
summary(anova.dataKE)

#ANOVA sqrt transformation
anova.dataKE <- aov(sqrt_CFU_mL ~ as.factor(Plasmid), data = dataKE)
summary(anova.dataKE)

#ANOVA cubrt transformation
anova.dataKE <- aov(cubrt_CFU_mL ~ as.factor(Plasmid), data = dataKE)
summary(anova.dataKE)

#ANOVA rec transformation
anova.dataKE <- aov(rec_CFU_mL ~ as.factor(Plasmid), data = dataKE)
summary(anova.dataKE)

#ANOVA sqrtrec transformation
```

```
anova.dataKE <- aov(sqrtrec_CFU_mL ~ as.factor(Plasmid), data = dataKE)
summary(anova.dataKE)

### Kruskal wallis test ###
kruskalKE <- kruskal.test(log_CFU_mL ~ Plasmid, data = dataKE)
summary(kruskalKE)
kruskalKE

### KW test post hoc dunn test ###
dunnKE <- dunnTest(log_CFU_mL ~ Plasmid,
                data = dataKE,
                method = "holm")
dunnKE

################## EK ################
#model
lmModEK <- lm(dataEK$log_CFU_mL ~ dataEK$Plasmid, data=dataEK) # initial model

par(mfrow=c(2,2)) # init 4 charts in 1 panel

plot(lmModEK)
#BP test
lmtest::bptest(lmModEK)  # Breusch-Pagan test
car::ncvTest(lmModEK)  # NCV test
#testing a different way to see if I get consistent results
fitEK <- lm(dataEK$log_CFU_mL~dataEK$Plasmid)
ncvTest(fitEK)

#view model summary
summary(fitEK)

#residuals
```

```
resEK <- resid(fitEK)
resEK

#produce residual vs. fitted plot
plot(fitted(fitEK), resEK)
#add a horizontal line at 0
abline(0,0)
#create Q-Q plot for residuals
qqnorm(resEK)
#add a straight diagonal line to the plot
qqline(resEK)
#Create density plot of residuals
plot(density(resEK))

#check heteroskedasticity
lmcEK <- lm(CFU_mL ~ Plasmid, data = dataEK)
gvlma(lmcEK) #assumptions met!!!

## ANOVA ##
# analysis of variance in EK conjugation
dataEK
anova.dataEK <- aov(CFU_mL ~ as.factor(Plasmid), data = dataEK)
summary(anova.dataEK)

# table with factors, means and standard deviation
dataEK_summary <- group_by(dataEK, Plasmid) %>%
    summarise(mean=mean(CFU_mL), sd=sd(CFU_mL)) %>%
    arrange(desc(mean))
print(dataEK_summary)

# Tukey's test
tukey.dataEK <- TukeyHSD(anova.dataEK)
```

```
print(tukey.dataEK)

# creating the compact letter display
cld.dataEK <- multcompLetters4(anova.dataEK, tukey.dataEK)
print(cld.dataEK)

# adding the compact letter display to the table with means and sd
cldEK <- as.data.frame.list(cld.dataEK$`as.factor(Plasmid)`)
dataEK_summary$Tukey <- cldEK$Letters
print(dataEK_summary)

### Kruskal wallis test log ###
kruskalEK <- kruskal.test(log_CFU_mL ~ Plasmid, data = dataEK)
summary(kruskalEK)
kruskalEK

### Kruskal wallis test nontransformed ###
kruskalEK <- kruskal.test(CFU_mL ~ Plasmid, data = dataEK)
summary(kruskalEK)
kruskalEK

### KW test post hoc dunn test ###
dunnEK <- dunnTest(log_CFU_mL ~ Plasmid,
        data = dataEK,
        method = "holm")
dunnEK

################## KK ################
#model
lmModKK <- lm(dataKK$log_CFU_mL ~ dataKK$Plasmid, data=dataKK) # initial model

par(mfrow=c(2,2)) # init 4 charts in 1 panel
```

```
plot(lmModKK)
#BP test
lmtest::bptest(lmModKK)  # Breusch-Pagan test
car::ncvTest(lmModKK)  # NCV test
#testing a different way to see if I get consistent results
fitEK <- lm(dataKK$log_CFU_mL~dataKK$Plasmid)
ncvTest(fitKK)

#view model summary
summary(fitKK)

#residuals
resKK <- resid(fitKK)
resKK

#produce residual vs. fitted plot
plot(fitted(fitKK), resKK)
#add a horizontal line at 0
abline(0,0)
#create Q-Q plot for residuals
qqnorm(resKK)
#add a straight diagonal line to the plot
qqline(resKK)
#Create density plot of residuals
plot(density(resKK))

#check heteroskedasticity
lmcKK <- lm(log_CFU_mL ~ Plasmid, data = dataKK)
gvlma(lmcKK) #assumptions met!!!

## ANOVA ##
```

```
# analysis of variance in KK conjugation
dataKK
anova.dataKK <- aov(log_CFU_mL ~ as.factor(Plasmid), data = dataKK)
summary(anova.dataKK)

# table with factors, means and standard deviation
dataKK_summary <- group_by(dataKK, Plasmid) %>%
    summarise(mean=mean(log_CFU_mL), sd=sd(log_CFU_mL)) %>%
    arrange(desc(mean))
print(dataKK_summary)

# Tukey's test
tukey.dataKK <- TukeyHSD(anova.dataKK)
print(tukey.dataKK)

# creating the compact letter display
cld.dataKK <- multcompLetters4(anova.dataKK, tukey.dataKK)
print(cld.dataKK)

# adding the compact letter display to the table with means and sd
cldKK <- as.data.frame.list(cld.dataKK$`as.factor(Plasmid)`)
dataKK_summary$Tukey <- cldKK$Letters
print(dataKK_summary)

### Kruskal wallis test log transformed ###
kruskalKK <- kruskal.test(log_CFU_mL ~ Plasmid, data = dataKK)
summary(kruskalKK)
kruskalKK

### KW test post hoc dunn test ###
dunnKK <- dunnTest(log_CFU_mL ~ Plasmid,
          data = dataKK,
```

```
        method = "holm")
dunnKK
```
